# A contextual approach for exploring faculty readiness to teach online

**DOI:** 10.1016/j.heliyon.2023.e20491

**Published:** 2023-10-13

**Authors:** Ghania Zgheib, Roula Al Daia, Mireille Serhan

**Affiliations:** a Department of Education, Faculty of Arts and Sciences, University of Balamand, Lebanon; b Faculty of Business and Management, University of Balamand, Lebanon; c Department of Nutritional Sciences, Faculty of Health Sciences, University of Balamand, Lebanon

**Keywords:** *Readiness to teach online*, *Course design*, *Attitudes towards online learning*, *Online pedagogy*, *Technology access and skills*, *Institutional support*

## Abstract

Effective online teaching and learning requires readiness of all stakeholders, students, faculty, and administrators while considering contextual factors that influence the design and delivery of online learning. The purpose of this study is to explore how ready for online teaching faculty are in a Lebanese higher education context and to investigate readiness factors that impact their online teaching. It also aims at identifying any significant differences in readiness based on gender, years of teaching experience and discipline. A survey was developed based on a review of the literature and existing surveys that address readiness factors for online teaching. The survey was administered in Spring 2020, and 210 faculty at a private higher education institution completed the survey. Exploratory Factor Analysis (EFA), Multivariate Analysis of Variance (MANOVA) and descriptive statistics were conducted. The EFA resulted in 5 factors associated with faculty readiness to teach online, namely technology access and skills, course design, online pedagogy, attitude, and institutional support. Descriptive statistics revealed that faculty have the minimum required technology skills to teach online, yet they are faced with technical challenges associated with the context and the need for institutional support. MANOVA tests revealed a statistically significant difference between female faculty members who are readier in terms of course design and attitude than their counterpart males, a statistically significant difference in terms of course design for faculty who have more years of teaching experience. As for discipline specific readiness, majors that are focused on art and design revealed to be less ready for online teaching. This study implies the need for a more robust infrastructure to expand the delivery of online learning in Lebanon and the need for professional development for faculty to create pedagogically and technically enhanced online courses.

## Highlights

• Factors associated with faculty readiness to teach online in a Lebanese context were identified.
• Factors that emerged in this study: Technology access and skills, course design, online pedagogy, attitude and motivation, and institutional support.
• Minimum requirements of technological readiness among faculty exist.
• Online teaching readiness varies with gender, years of teaching experience and discipline.

## 1 Introduction

In the wake of the COVID-19 pandemic that left the world no choice but to transition face-to-face daily operations to online, educational institutions around the world experienced this disruption as well and shifted to online learning without any preparedness. This all-of- a-sudden shift has certainly saved the academic year for students and higher education institutions that did not have to shut down [1,2]. However, as opposed to conventional transitioning to online teaching, it turned out to be crisis online teaching [3,4]. Several challenges such as communication, online assessments, use of technology, experience with online teaching, mental health and time management have been associated with the shift to online education during the pandemic [5].

Online learning requires advanced technology skills and modified teaching methods compared to face-to-face teaching [6,7]. Hence the level of readiness of faculty to transition to online teaching is an indicator of success or failure in this area. Readiness to teach online needs to consider technical skills, pedagogical knowledge, communication skills as well as the culture and structural differentials that promote equitable experiences [1].

While online course delivery has been there for a long time, not all countries have policies that govern the offering of online courses, and some countries had never even explored online teaching before the pandemic. In the Middle East and in Lebanon in particular, online learning had been an underexplored area until all educational institutions were forced to shift to online teaching due to COVID-19 [8]. Until now, there are no guidelines and policies that govern online learning in Lebanon despite a draft law that has been proposed in May 2016 allowing a maximum of 60 % of a course to be delivered online which has not taken effect yet [9].

Both students' readiness to learn online and faculty readiness to teach online are key in the success of online learning. In a survey conducted to investigate students' readiness to learn online in a private higher education institution in Lebanon, Zgheib et al. [10] revealed that higher education students in Lebanon possess the necessary technology skills, have access to technology, have communication and social interactions needed in online environments, and self-regulated skills to learn in online environments. This study contradicted findings by Tarhini et al. [11] who revealed that students’ adoption of eLearning is immature among Lebanese students as a result of the traditional pedagogies used in instruction and technology issues. Similary, El Turk and Cherney [12] confirm that structural, pedagogical and technical issues had been barriers to online learning adoption in Lebanon.

While Zgheib et al. [10] suggested that students are ready to learn online, the pandemic put institutions to the test and more specifically faculty who had to develop technology skills and online pedagogies in a very short time. Previous research has shown that faculty have yet to exploit the affordances of technologies and need support to implement technology in their teaching [13]. In addition to faculty skills, Lebanon faces infrastructure challenges related to internet speed, electricity, and access to personal devices that facilitate the online learning process [8]. Regardless of the challenges associated with online learning around the world, the transition to online learning during the pandemic has contributed to the development of digital literacy and autonomous learning of students [14].

Given the fact that online education is new in the educational field in Lebanon, little research has been conducted in this area making it an under-researched area. The present study aims at capitalizing on the importance of readiness to teach online in crisis and under normal circumstances in order to inform policies and guidelines for quality online learning. Since higher education faculty are main stakeholders in the area of online education and context plays a key role in identifying readiness to adopt online teaching, this study examines factors influencing faculty readiness to adopt this mode of teaching, validating and confirming factors associated with online teaching readiness in a Lebanese context, and investigating the readiness of faculty in this area.

## 2 Literature review

### 2.1 Factors influencing faculty readiness to teach online

**Fig. 1:**
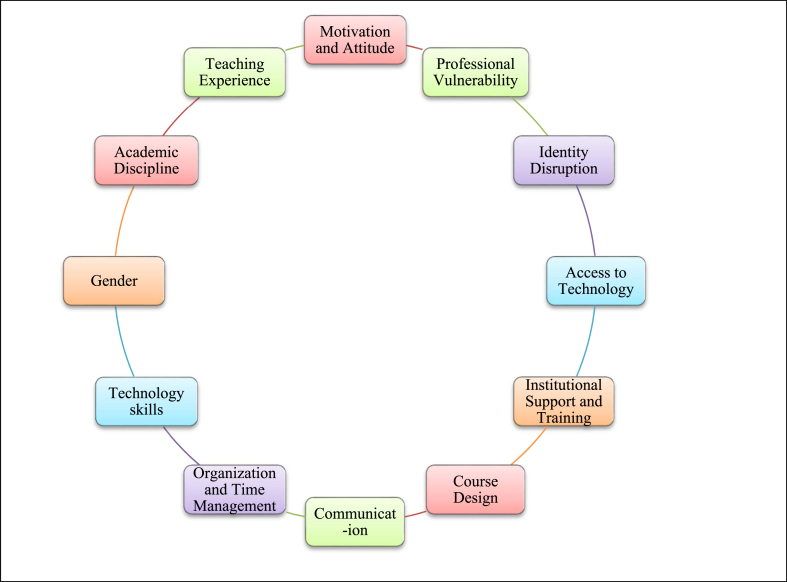
Factors associated with faculty readiness to teach online based on the literature.

Faculty readiness to teach online is defined as their preparedness to develop and implement online instruction [3,15]. Online surveys related to the assessment of online teaching readiness such as UC Davis's survey, and University of Toledo's survey and the literature, pre-dominantly regard faculty attitudes, institutional support, technology skills, time management skills, communication and course design as the main factors in identifying readiness for online instruction. Generally speaking, course design, communication competence, time management, and technological competence appeared to have played a central role in online teaching readiness [16]. In recent research conducted during the pandemic, Cutri and Mena [1] and Cutri et al. [3] took into account the affective domain and institutional cultures that put more emphasis on research than on teaching as additional factors that contribute to faculty readiness to teach online. However, readiness factors are compromised when we look at temporary shift to online teaching [3]. An overview of factors associated with faculty readiness for online instruction is presented in Appendix A, elaborated in the literature review below, and synthesized in Fig. 1.

#### 2.1.1 Motivation and attitude

Attitude towards online learning determines faculty's adoption or resistance to this mode of instruction [17,18]. Based on the literature, attitude may be determined by different factors such as self-efficacy beliefs regarding online teaching [18,19] time needed to develop online courses [18], experience [20], peer pressure [18], and technical support [21,22].

#### 2.1.2 Professional vulnerability and identity disruption

Research suggests that there is an identity shift that faculty experience when shifting to online teaching. This disruption is mainly on the expertise level [23,24], and on the researcher identity level [25]. Faculty members feel threatened in online learning environments as the power dynamics shifts and they are no longer the deliverers of information. At the same time, they have to develop a new area of expertise which is online course delivery [3]. This area of expertise comes at the expense of faculty research where they find themselves having to invest time in the development of online courses and delivering them. This also results in professional vulnerability [25].

Similarly, faculty experience professional vulnerability since online learning introduces new practices and challenges to faculty who are experts in their own fields, faculty feel threatened [1]. Professional vulnerability is defined as “the feeling that one's professional identity and moral integrity, as part of being ‘a proper teacher’, are questioned and that valued workplace conditions are thereby threatened or lost” [26, p. 319]. Vulnerability is inseparable from tenure reviews which take into considerations teaching practices. Vulnerability could lead to lack of confidence in online teaching, something that could negatively affect teacher's performance, as confidence in online teaching was found to be a determining factor in online teaching performance [[26], [27]].

#### 2.1.3 Access to technology

Online teaching and learning rely on internet and a computer which are the primary instruments for learning [28]. The main infrastructure issues associated with online learning in Lebanon include the bad internet connection, the cost of internet connection, power cuts, and lack of technological devices jeopardizing the successful implementation of online learning [8,29].

#### 2.1.4 Institutional support and training

Online teaching requires the knowledge of synchronous and asynchronous software, how to troubleshoot them, how to assist students, and how to develop digital content [30,31]. A main component of a successful online teaching experience is having the technology skills needed to design and deliver online content. In some cases, teachers possessed technical competencies for online teaching but were found to be lacking on the front of course design, communication and time management competencies [32].

Support in transitioning to online teaching is vital for its success [33]. As importantly, the leadership's attitude towards online learning influences the motivation of faculty to implement online learning [34]. The quick transition to online teaching during the pandemic resulted in limited technical and pedagogical support for online course design and implementation in higher education [35]. Gay [36] emphasizes the importance of providing online help desk support for faculty to enhance their overall online teaching experience. Therefore, technical skills are essential, nevertheless they should not be dissociated from their pedagogical underpinnings.

#### 2.1.5 Course design

Like face-to-face course design, online course design requires careful alignment between objectives, content, activities, assessment, material, and student interaction [15]. Most experienced online faculty use a systemic instructional design approach to design their online courses by chunking the course into modules or weekly topics setting clear objectives and expectations for every week creating a roadmap for their learners [31,37]. Effective online course design also emphasizes creating a learner-centered environment scaffolded by presenting content in different formats to cater to the learners’ needs and promote student interaction through multiple outlets of expression [31].

#### 2.1.6 Communication

A key component to engage students in online courses is effective communication [38]. Communication in online courses starts from the syllabus and extends to online teaching. Faculty are expected to communicate clearly course expectations before the course begins and to be present during the course by sharing announcements, participating in online discussions, and providing feedback to students in order to foster learner-content, learner-learner, and learner-instructor interaction [31,39,40]. Faculty need to be familiar with the right online communication strategies to promote student engagement in online courses [15].

#### 2.1.7 Organization and time management

The ubiquity of online courses blurs the boundaries between class time and non-class time thus creating a time management issue for faculty [41]. The workload that results from managing grading, responding to students, and participating in discussion posts is overwhelming to online faculty [41]. Managing time effectively is a contributor to successful online teaching [42], and faculty may accumulate this skill through years of teaching experience, levels of institutional support, and technical support [43].

#### 2.1.8 Gender

Research on gender as an indicator of online teaching readiness is inconclusive [44]. Some research showed that female professors in higher education have reported higher self-efficacy in designing and implementing online courses [31,45], building an online program community [46], communication and time management [31], overall readiness [47]. Scherer et al. [47] also pointed out that while readiness levels may differ across genders, they also differ within gender as a result of different contextual factors such as taking into account online teaching during the pandemic. Scherer et al. [48] have uncovered, quantified, and explained the gender differences in readiness for online learning in an

international sample of higher-education teachers; results show gender differences exist but vary

in both direction and size across readiness constructs.

#### 2.1.9 Academic disciplines

Disciplines have their own specificities regardless of online learning which in turn calls for pedagogical content knowledge [47]. Research on online teaching and learning across different disciplines suggests some differences in terms of readiness profiles [46,47]. Faculty beliefs in online teaching and learning as well as their beliefs in their disciplines may contribute to their readiness to teach online [49].

#### 2.1.10 Teaching experience

Readiness to teach online is associated with the faculty experience in online teaching; the more experience the faculty member has, the more self-confident he/she is to teach online [22,31]. This result was recently confirmed by Bolliger and Halupa [27]. Furthermore, Scherer et al. [50] have found robust evidence that faculty's readiness for online learning increased first and then decreased with more experience; experienced instructors in higher education could benefit more from experience-appropriate, pedagogical, and content-related support programs for online learning.

## 3 The present study

Faculty readiness to teach online varies according to culture, country, and organization [15]. Therefore, there is a need to study online teaching readiness in different contexts and under different circumstances in order to provide contextual support for online teaching. The purpose of this study was to validate a comprehensive survey that was designed to allocate factors associated with instructors’ readiness to teach online in a Lebanese higher education context. Also, the study aimed at revealing the readiness of faculty to teach online in an emergency situation. The overarching research question was: “What factors of the Online Teaching Readiness Survey (OTRS) inform the readiness of faculty to teach online in a Lebanese Higher Education context?”, in addition to the following two research questions.

1. How ready for online teaching are faculty in a Lebanese Higher Education Institution?
2. How does online teaching readiness compare across gender, years of teaching experience, and faculties?

## 4 Methodology

### 4.1 Instrument

A survey instrument was developed to elicit instructors' readiness to teach online in the context of a Middle Eastern higher education institution. Institutional approval to conduct this study and to disseminate it to the university community was obtained from the office of the Provost. The survey was shared with the faculty through the institution's official email list and via an announcement email sent on behalf of the office of the Provost. By completing the online survey, the faculty had provided their consent to participate in this study. The items in the survey were based on an extensive literature review that examined faculty readiness to teach online (Appendix A) [15,[51], [52], [53], [54], [55], [56], [57]]. The items in the different instruments were gathered and analyzed to identify common themes and patterns resulting in our survey which included a set of 46 statements in reference to a five-point Likert scale (ranking from 1 “Strongly Disagree” to 5 “Strongly Agree”). The complete survey can be found in Appendix B. Likert-based instruments are the tools of choice to elicit online teaching readiness as shown in several previous studies and identify the factors underlying teaching readiness in an online environment [15,[51], [52], [53], [54]]. The survey was conducted during the Spring Semester of the 2019–2020 academic year, a few weeks after the first lockdown entered in force in Lebanon. The survey instrument was sent electronically to all full-time and part-time faculty members through Google Forms.

### 4.2 Context and participants

The study was conducted at a Middle Eastern higher education private institution in Lebanon. There are currently more than 1300 faculty members in total. A follow-up reminder e-mail was sent to elicit more responses and the final number of respondents reached 210, representing around 16 % of the entire faculty population.

### 4.3 Data analysis

Descriptive statistics (means and standard deviations) were reported both at the item

level, at the subscale level, and also by various demographic factors. An exploratory factor analysis (EFA) was conducted to identify the factors underlying instructors’ readiness to teach online. Multivariate analysis of variance (MANOVA) was conducted to elicit variations in online teaching readiness according to significant demographic variables collected as part of the survey. The chosen independent variables were gender, the faculty of affiliation and years of experience in line with similar studies conducted in other contexts.

## 5 Results

### 5.1 Descriptive statistics

A total of 210 faculty members responded to the survey, of whom 115 (54.8 %) were female and 95 (45.2 %) were male. The largest percentage of responses was collected from instructors (34.3 %), while assistant, associate and full professors responded at the following rates, 24.3 %, 14.8 % and 6.7 % consecutively. The highest percentage of respondents (40 %) are between 31 and 40 years old. With reference to the faculty affiliation, 33.3 % of the respondents are from the Faculty of Arts and Sciences, followed by Health Sciences (17.6 %), Engineering (17.1 %), Faculty of Technology (13.3 %), Business and Management (9.5 %), and Fine Arts (7.11 %); the sample also included 1 % of Medicine and Medical Sciences, and 1 % of Institute of Theology. Most of the respondents (61.9 %) were full timers. More than half of the respondents (56.2 %) had more than 10 years of teaching experience. Of the faculty who participated in this study, 80 % of them had less than 6 months of online teaching experience. Appendix C presents a description of the respondents, including age, gender, faculty affiliation, rank, academic status, years teaching, and years teaching online.

Descriptive analysis is presented in Table 1 and indicates a high level of technological readiness in terms of access to equipment. More than 90 % of the respondents have personal computers or laptops, have access to a webcam and a microphone that can be used for videoconferencing. Many respondents have the basic skills to operate computers (82.4 %), to send and receive emails and open and send email attachments (more than 90 %) and to use basic internet functions (84.3 %), however, over 50 % of the respondents don't have the sufficient skills to create online tests and assignments and to use advanced features of Moodle or any other learning management system. About 73 % agreed that they can provide a comprehensive syllabus that adheres to the institution's policy and they can design assignments that assess the course learning outcomes. More than 50 % of the respondents reported that they can design learning activities that provide students opportunities for interaction in an online environment and the ability to develop an online course.

**Table 1:** Descriptive statistics of survey responses by item (expressed in %).

*Faculty readiness competencies*	Strongly Disagree	Disagree	Neither Agree Nor Disagree	Agree	Strongly Agree	WA^a^±SD^a^, ^b^
1. I have access to a personal computer or laptop	1	0	0.5	9	89.5	**4.86 ± 0.49**
2. I have access to a printer	21	14.3	11.4	17.6	35.7	**3.33 ± 1.57**
3. I have access to a scanner	22.4	16.7	8.6	16.2	36.1	**3.27 ± 1.61**
4. I have access to a webcam and a microphone that I can use for video conferencing	1.4	2.4	2.9	14.3	79	**4.67 ± 0.77**
5. I have access to reliable internet connection when needed	1.9	13.8	17.6	33.8	32.9	**3.82 ± 1.09**
6. I can complete basic computer operations	0.5	1	2.4	13.8	82.4	**4.77 ± 0.58**
7. I can use basic internet functions	0.5	0.5	1	13.8	84.3	**4.81 ± 0.51**
8. I can send and receive emails and open and send email attachments	0.5	0.5	0.5	6.2	92.4	**4.90 ± 0.43**
9. I have the skills to create instructional videos	2.9	4.3	15.2	29.5	48.1	**4.16 ± 1.02**
10. I have the skills to create online tests and assignments	6.7	11	31.4	21.4	29.5	**3.56 ± 1.20**
11. I can use web-conferencing tools	0.5	0	3.3	22.4	73.8	**4.69 ± 0.58**
12. I can use basic features of Moodle or any other Learning Management System	0.5	3.8	5.7	16.7	73.3	**4.59 ± 0.80**
13. I can use advanced features of Moodle or any other Learning Management System	4.3	13.8	26.7	27.1	28.1	**3.61 ± 1.15**
14. I can provide a comprehensive syllabus that adheres to my institution's policy.	0.5	0	2.4	24.3	72.9	**4.69 ± 0.56**
15. I can write measurable learning outcomes and specific course objectives	0.5	1	3.3	27.1	68.1	**4.61 ± 0.64**
16. I can design assignments that assess my course learning outcomes.	0.5	0	3.3	23.3	72.9	**4.68 ± 0.58**
17. I can revise course content and instructional materials based on student feedback, as needed	0.5	1	4.3	21.9	72.4	**4.65 ± 0.65**
18. I can design learning activities that provide students opportunities for interaction in an online environment	1.4	7.1	17.1	34.8	39.5	**4.04 ± 0.99**
19. I am able to develop an online course	1	3.3	24.3	37.6	33.8	**4.00 ± 0.89**
20. I lecture to my students for most of the online class session	2.9	14.3	13.3	32.4	37.1	**3.87 ± 1.14**
21. I provide timely and constructive feedback on students' assignments	1.4	1	11.9	41	44.8	**4.27 ± 0.81**
22. I use non-traditional assessments	2.4	7.1	16.2	30.5	43.8	**4.06 ± 1.04**
23. I send reminders to students about upcoming assignments	3.3	2.9	14.3	28.1	51.4	**4.21 ± 1.01**
24. I can use online discussion as a means of teaching	1.9	2.4	9.0	39.0	47.6	**4.28 ± 0.87**
25. I can use online chat function as a means of teaching.	4.8	4.3	10.5	33.3	47.1	**4.14 ± 1.07**
26. I use a variety of teaching strategies to promote online interaction among my students	1.9	2.9	11.4	40	43.8	**4.21 ± 0.89**
27. I can accommodate students that have accessibility issues	1.9	7.1	22.4	35.2	33.3	**3.91 ± 1.00**
28. I can continuously monitor and manage student progress by using course statistics or reports on Moodle	5.2	11.9	26.7	32.9	23.3	**3.57 ± 1.12**
29. I communicate effectively and comfortably in writing using email or text messaging	0.5	0.5	1.4	20	77.6	**4.74 ± 0.55**
30. I respond to students' questions promptly	0.5	0	1	18.6	88	**4.78 ± 0.50**
31. I can communicate expectations about my students' online behavior in my course	1.4	2.4	25.7	38.6	31.9	**3.97 ± 0.89**
32. I feel comfortable conveying my personality and/or emotions through speaking	0.5	1.9	12.9	35.7	49	**4.31 ± 0.80**
33. I find that online teaching takes more time than face-to-face instruction	2.4	7.1	10.5	28.6	51.4	**4.20 ± 1.04**
34. I am good at organizing teaching materials into modules, weeks, topics, or units	0.5	0	6.2	37.1	56.2	**4.49 ± 0.65**
35. I usually schedule time to design the course prior to delivery	1	3.8	13.8	42.4	39	**4.15 ± 0.86**
36. I have the time to develop an online course	4.3	8.1	29.5	39	19	**3.60 ± 1.02**
37. I use features in Moodle or another tool in order to manage time	8.6	14.3	30	28.1	19	**3.35 ± 1.18**
38. I am open to learning more ways in using technology for teaching	0.5	0	5.7	21.9	71.9	**4.65 ± 0.63**
39. I believe teaching online offers greater course flexibility for students	4.8	10.5	31.9	29.5	23.3	**3.56 ± 1.10**
40. I believe teaching online diversifies program offerings	3.3	11.9	25.7	35.2	23.8	**3.64 ± 1.07**
41. I believe teaching online offers more opportunities to learn new technologies and improve my teaching	2.4	5.7	21.9	34.8	35.2	**3.95 ± 1.00**
42. I believe online learning is able to promote the students' acquisition of skills	5.2	13.3	25.7	34.8	21	**3.53 ± 1.12**
43. I am motivated to teach online	2.4	4.3	19	42.9	31.4	**3.97 ± 0.94**
44. I believe my university supports innovative teaching methods	2.9	2.4	9.5	39.5	45.7	**4.23 ± 0.92**
45. I have persons and/or resources at my university who will assist me with any technical problems I might have with my software applications as well as my computer hardware	1.9	3.3	11	44.3	39.5	**4.16 ± 0.88**
46. I need more training and guidance to implement online learning in my courses	5.7	15.2	27.6	36.7	14.8	**3.40 ± 1.08**
						

a Weighted Average.

b Standard Deviation.

Many respondents (more than 80 %) use online discussion as a means of teaching, provide timely and constructive feedback on students' assignments, send reminders to students about upcoming assignments and use a variety of teaching strategies to promote online interaction among students. Lowest scores relate to lecturing to students for most of the online class session (69.5 %) and continuously monitoring and managing students' progress by using course statistics or reports on Moodle (56.2 %). The findings are regarded as compatible with the respondents’ technical skills.

With respect to items related to communication, nearly all participants can respond to students' questions promptly (more than 95 %), while 70 % of the respondents can communicate effectively and comfortably in writing using email or text message, feel comfortable conveying personality and emotions through speaking and communicate expectations about students’ online behavior in the course.

The following competencies were rated the highest among the respondents (more than 80 %): organizing teaching materials into modules or units, finding that online teaching takes more time than face-to-face instruction and scheduling time to design the course prior to delivery. However, the following competencies were rated the lowest: about 58 % of the respondents agreed they have time to develop an online course and nearly 47 % confirmed using features in Moodle or another tool in order to manage time. Although most respondents believe that the academic institution supports innovative teaching methods (more than 85 %), however, they need more training and guidance to implement online learning in the courses and assistance with technical problems.

### 5.2 Factor analysis

Exploratory factor analysis (EFA) was used to identify the factors underlying instructors' readiness to teach online. The initial scale of 46 items exhibited a high level of reliability with an overall Cronbach alfa of 0.919. Out of the initial 46 items included in the original instrument, the final EFA resulted in 30 items. To reach this final number of items, several steps were followed. Variables exhibiting less than 20 % of correlations above the threshold of 0.3 were excluded, while one item was excluded on the basis of negative total item correlation. Other items exhibited less than 20 % of correlations above 0.3; they were however kept in the analysis due to their importance in eliciting online teaching readiness and because the authors deemed to have no close substitutes in the other statements. Additional items were excluded due to cross-loadings or because of absence of loading altogether. The EFA was conducted using varimax rotation. The Kaiser-Meyer-Olin measure for sampling adequacy took a value of 0.867, well exceeding the 0.5 threshold recommended by Kaiser (1974). The Chi square of Bartlett's test of sphericity was found to be equal to 3852.673 (p < 0.001), showing the existence of an acceptable level of collinearity between variables in the inter-correlation matrix.

**Fig. 2:**
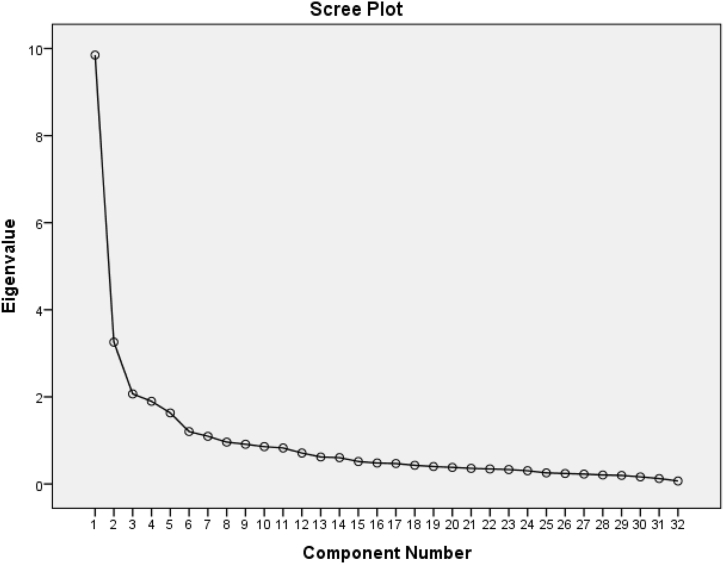
Scree Plot for Faculty online learning readiness survey.

The scree plot (Fig. 2) was used as the tool for determining the final number of factors which appears to be at five, the Kaiser Eigen value criterion would have led to the adoption of seven factors with a total variance explained of 65.64 %, versus 58.45 % of total variance explained for five factors; however, based on the literature as well as on researcher's judgement it was decided to opt for a five-factor structure as it appeared more coherent than a seven factor structure.

Table 2 shows the grouping by factors as identified by the researchers based on the labeling of common item themes. The overall scale exhibits a high level of reliability with a Cronbach's alfa of 0.919 showing a high level of internal consistency. In addition, reliability was verified for each factor, and each of the five factors identified below was found to possess adequate level of reliability as measured by the sub-component Cronbach's alfa.

**Table 2:** Factors identified by the EFA and underlying components.

Rotated Component Matrix					
	Component				
	1	2	3	4	5
**Technology Access and Skills (Cronbach's α = 0.828)**					
I can complete basic computer operations (e.g., creating and editing documents, managing files etc)	.759				
I can use basic internet functions such as web browsing, uploading and downloading documents and locating resources.	.748				
I can use basic features of Moodle or any other Learning Management System (file upload, editing, enrolling users …)	.707				
I can use web-conferencing tools (e.g. Webex, Skype) for teaching.	.705				
I have access to a webcam and a microphone that I can use for video conferencing.	.694				
I have access to a personal computer or laptop.	.592				
I have the skills to create instructional videos (e.g. lecture video, demonstrations, video tutorials).	.549				
I can use advanced features of Moodle or any other Learning Management System (online grading, creating discussions, linking learning outcomes to assignments, monitoring analytics, …).	.467				
I have access to reliable internet connection when needed.					
**Course Design (Cronbach's α = 0.862)**					
I can provide a comprehensive syllabus that adheres to my institution's policy.		.807			
I can write measurable learning outcomes and specific course objectives.		.791			
I can design assignments that assess my course learning outcomes.		.760			
I am good at organizing teaching materials into modules, weeks, topics, or units.		.702			
I can revise course content and instructional materials based on student feedback, as needed.		.688			
I usually schedule time to design the course prior to delivery (eg. A month before delivery).		.570			
**Online Pedagogy (Cronbach's α = 0.857)**					
I use non-traditional assessments (projects, papers, case studies, reflections) to assess my students' learning.			.699		
I can use online discussion as a means of teaching.			.679		
I use a variety of teaching strategies to promote online interaction among my students.			.665		
I provide timely and constructive feedback on students' assignments.			.654		
I send reminders to students about upcoming assignments.			.627		
I am able to develop an online course.			.534		
I can design learning activities that provide students opportunities for interaction in an online environment (e.g., discussion forums, wikis).			.525		
I can communicate expectations about my students' online behavior in my course (Netiquette).			.492		
I feel comfortable conveying my personality and/or emotions through speaking (audio/video).			.467		
**Attitude Towards online teaching (Cronbach's α = 0.887)**					
I believe teaching online diversifies program offerings.				.870	
I believe online learning is able to promote the students' acquisition of skills (e.g. communication skills, computer skills, problem solving skills, etc).				.834	
I believe teaching online offers greater course flexibility for students.				.821	
I believe teaching online offers more opportunities to learn new technologies and improve my teaching.				.792	
I am motivated to teach online.				.720	
**Support (Cronbach's α = 0.726)**					
I believe my university supports innovative teaching methods.					.769
I have persons and/or resources at my university who will assist me with any technical problems I might have with my software applications as well as my computer hardware.					.746
I have the time to develop an online course.					.601

#### 5.2.1 Five factors emerged as a result of the EFA

*Technology access and skills:* This first factor reflects how well instructors appear to be equipped in terms of hardware as well as the skills displayed when it comes to its use as highlighted in the descriptive statistics. Peripheral equipment levels are lower. The latter however are increasingly being perceived as secondary, especially that they do not affect the way online instruction is imparted. In addition, in terms of basic computer and internet functions, proficiency levels are nearly perfect especially in terms of basic computer operations, basic internet operations and sending and receiving e-mail. Percentages fall significantly when it comes to using advanced features of Moodle (55.2 % agree or strongly agree).

*Course design:* The second factor labelled “course design” elicits general pedagogical skills, and features traditional items related to writing and measuring course learning outcomes and course preparation skills. instructors appear to be well prepared on this front (see descriptive statistics for “I can write measurable learning outcomes and specific course objectives” and “I can design assignments that assess my course learning outcomes”). Overall, faculty members in this institution appear to be very proficient in fundamentals of course design and measurement of course outcomes and objectives.

*Online pedagogy:* Two items in particular called our attention with respect to this factor. They are labelled “I use non-traditional assessments (projects, papers, case studies, reflections) to assess my students' learning” and “I send reminders to students about upcoming assignments”. We find these two items to be closely linked to this factor for their specific relevance as non-traditional teaching methods. In addition, the other elements of this component highlight promising dispositions on behalf of the teachers, whether in terms of strategies used to promote online interaction or conveying one's personality in online environments.

*Attitude toward online teaching:* This factor includes items such as “I believe teaching online diversifies program offerings”, “I believe online learning is able to promote the students' acquisition of skills (e.g. communication skills, computer skills, problem solving skills, etc),” “I believe teaching online offers greater course flexibility for students,” “I believe teaching online offers more opportunities to learn new technologies and improve my teaching.” Interestingly, percentage responses show a dissonance between the perception of online teaching and learning's benefits for learners versus instructors', with the latter considering online teaching as self-improving, while questioning its effectiveness from learner's perspective.

*Institutional* support*:* The final factor is related to institutional support and includes three factors: the first two relate to university support while the third refers to time availability. While there seems to be wide consensus regarding availability of support for online learning activities, time and resources appear to be scarce when it comes to developing online courses.

### 5.3 MANOVA

Variations in terms of gender, faculty of affiliation and years of teaching experience were examined in line with similar studies conducted in other contexts. Table 3 provides average scores for each of the identified EFA factors, followed by the MANOVA analysis for each of gender, faculty of affiliation and years of teaching experience.

**Table 3:** Average scores by Factor.

	Technology Access and Skills	Course Design	Online Pedagogy	Attitude Towards Online Teaching	Support
Gender Averages					
Female	4.51	4.61	4.20	3.87	4.06
Male	4.53	4.46	4.09	3.56	3.92
**Years of Experience Averages**					
>10 years	4.52	4.60	4.17	3.72	3.97
7–10 years	4.55	4.64	4.17	3.61	4.05
4–6 years	4.49	4.34	4.03	3.99	4.12
1–3 years	4.49	4.30	4.21	3.59	3.79
**Faculty of Affiliation Averages**					
Arts and Architecture	4.07	4.27	3.96	3.23	3.80
Arts and Sciences	4.59	4.59	4.14	3.62	3.99
Business and Management	4.65	4.58	4.27	3.91	3.75
Engineering	4.53	4.50	4.12	3.69	3.99
Health Sciences	4.42	4.64	4.11	4.10	4.13
Medicine	4.31	5.00	4.28	5.00	4.33
Technology	4.61	4.43	4.24	3.56	4.14
Theology	4.63	4.75	4.61	4.40	3.83

#### 5.3.1 Faculty affiliation

The Higher Education Institution where the survey was conducted is home to seven faculties, covering a comprehensive array of specializations (Arts and Sciences, Business and Management, Engineering, Medicine, Medical Sciences, Technical Degrees, Theology, Architecture and Fine Arts). In this context, the MANOVA followed by post-hoc testing reveals differences in terms of readiness to teach online, and whether some faculties might have been expanding further effort to build capacity when it comes to online teaching and learning.

In this respect, the MANOVA test showed that there was a statistically significant difference in readiness based on faculty of affiliation, F (35, 835) = 1.881, p < 0.005; Wilk's Λ = 0.727, partial η2 = 0.062. Pillai's Trace = 0.301, F(35, 1010) = 1.848, p = 0.002, partial η2 = 0.060.

When it comes to each component of the readiness instrument, it was found that affiliation made a difference when it came to Access to Technology F = 2.437, df(7), p = 0.02 and Attitude F = 3.096, df(7), p = 0.004. This may be linked to the differences in teaching and learning methodologies used across disciplines therefore requiring different typologies of interactions and tools in online environments.

Post hoc tests were also conducted (Tukey HSD test), and they show the following. Readiness for online teaching with respect to the access to technology dimension of Architecture and Fine Arts faculty was found to be significantly lower than that of the Arts and Sciences, Business and Management and the Technology Institute. This can be attributed to the fact that education in the creative field, requires knowledge to be imparted using different methodologies from the ones used on more classical fields. In terms of Attitude Towards Online Teaching, only one difference was highlighted, and that was a significantly lower score for the Faculty of Architecture and Fine Arts relative to the Faculty of Health Sciences.

#### 5.3.2 Gender

A MANOVA was used to compare the readiness of male and female instructors across all dimensions of online learning. The multivariate result was significant for gender: Pillai's Trace = 0.066, F = 2.893, df = (204), p = 0.015. We use Pillai's Trace due to the fact that Box's M test was found to be significant.

The univariate F-tests show a significant difference between men and women for Course Design, F = 4.193, df = (1), p = 0.042; and Attitude Towards Online Teaching F = 6.891, df = (1), p = 0.009. More generally, the results revealed that female faculty members exhibited stronger readiness than their male counterparts on these two dimensions.

#### 5.3.3 Years of experience

The third MANOVA attempted to elicit differences according to the number of years of teaching experience, with results confirming the existence of such an influence on readiness to teach online F (15, 558) = 1.709, p < 0.05; Wilk's Λ = 0.883, partial η2 = 0.040. Pillai's Trace = 0.120, F = 1.699, df = 15, p = 0.047. However, this result was only validated at the level of course design, which was confirmed with a post hoc test showing that a larger number of years of experience was conducive to more readiness when it came to course design.

## 6 Discussion

### 6.1 Readiness of faculty to teach online in a Lebanese Institution

With respect to technical skills, although many respondents (more than 82.4 %) have the basic skills to operate computers, however, over 50 % of the respondents do not have the sufficient skills to create online tests and assignments and to use advanced features of Moodle or any other learning management system. Technical skills have a strong impact on faculty readiness to teach online. The literature has revealed that the more familiar the instructors are with technology, the readier they are for teaching in an online setting [17]. According to Ncube et al. [58], the fast progress of technology might become challenging for instructors as technology could be intimidating. Furthermore, one of the utmost important factors influencing instructor online teaching readiness is the online pedagogy. It is clear that the majority possesses some online teaching skills (83.8 % agree or strongly agree about their ability to use a variety of strategies to promote online interaction; whereas 84.7 % agree or strongly agree that they feel comfortable conveying their personality online). In parallel, many faculty do not know how to address accessibility issues and track students’ progress; around a third are not very savvy when it comes to LMS use. When assessing the impact of this factor, Gay [53] evaluated whether online instructors prefer a traditional classroom setting compared to an online environment; results revealed the characteristics of online instructors that are suitable for the online environment. Online teaching requires the shift from traditional teaching methods to innovative ones where instructors need a set of online content and resources to facilitate the learning process.

With reference to course design and the characteristics of the instructor, the majority possesses traditional course design skills (95 % can write measurable learning outcomes and 96 % can design assessments to measure them) while several reported challenges in developing an online course and creating interactive online activities. Liu et al. [59] found that more years of teaching experience instructors had, the more familiar they would typically be with educational practices relating to technology-based teaching and learning. Faculty reported having enough support from their institution in terms of pedagogy and technology; however, only 58 % agree or strongly agree that they have the time to develop an online course. Many studies have emphasized the importance of training programs needed to support the instructor e-readiness. Such programs should be varied in different aspects of online teaching, including technical skills, online teaching methodology and pedagogy and online educational content [58]. Finally, time constraint was also an element influencing instructor readiness for e-learning. However, it was not widely expressed in the literature and its role compared to other factors is less crucial. Koo [60] reported that time constraint could be a major challenge to affect the instructors’ perceived readiness for online collaborative learning.

### 6.2 Factors of online teaching readiness in a Lebanese Higher Education Institution

While the respondents demonstrated a more than adequate level of access to technology, the importance of this element has been emphasized in the early years of development of online learning as an important element to be elicited when assessing readiness for e-learning [28]. Elements of access to technology had been incorporated into earlier surveys aiming to elicit readiness to teach online [51,53]. This concern remains relevant today in the context of developing countries where access to technology and the underlying infrastructure remain lacking [29]. In more recent contributions, this dimension is not investigated; the emphasis is rather put on technical competence. In other words, the competences needed for the use of technology and not access to computers, printers and other terminals per se [15].

In terms of course design, the faculty's self-assessment shows confidence in their mastery over the fundamentals of course design, in line with other surveys [15]. This element is considered a steppingstone and a sin *qua non* condition before even considering readiness for online teaching. Actually, mastery over course design fundamentals is at the heart of the teaching profession and predates the third factor identified in the EFA and which is related to online pedagogy. On this front, the results appear promising with most faculty members. Some of the elements on online pedagogy can be found in other surveys under course communication [31]; the importance of the online persona of the teacher via the adaptation to new pedagogies is central to the online learning process [17]. Nevertheless, this dimension needs to be nurtured and enhanced through in-house training and development especially that the above results are based on the perception of instructors themselves and need triangulation for validation. In reality, course design needs to become a priority in online learning environment to avoid some of the pitfalls generated by the ubiquity of the online classroom [60].

Based on the highlighted components of online pedagogy as laid out in Table 2 and it implies the necessity for a “reset” on the teachers’ behalf in terms of the acquisition of new online reflexes, which do not always merely represent a transposition of class practices to an online environment. For instance, the rules of class discussions and the nature of interactions it necessitates are not the same whether in an online setting or a traditional setting. This requires that the teacher should overcome anxiety caused by the use of new technology and to become a creative professional [61]. Moreover, the component that relates to communicating expectations plays a central role in securing comfortable online learning environments [41].

In terms of attitude, it is important to highlight the affective dimension of the process. For instance, faculty's emotional response may condition the process of transition to online learning and needs to be acknowledged and addressed in order to avoid emotional resistance and identity disruptions [1]. In this perspective, optimism [3] may have been a driver of the motivation to teach online, with 74.3 % of respondents agreeing and strongly agreeing to the statement: “I am motivated to teach online).

Similar factors have been highlighted in other studies especially in terms of institutional support and pedagogy (learning-transfer self-efficacy) [62]. In addition, attitude and institutional support are strongly intertwined. The latter is fundamental since acquiring new skills is only one part of the process; another part pertains to the affective process and insecurities that instructors might suffer from and their need to develop a sense of belonging in new virtual environments [63] that will allow them to overcome their anxiety to teach online [62]. In a nutshell, attitude is key [3] and institutions have a responsibility to guide their faculty members throughout the process in technical, pedagogic but also through emotional and affective support.

### 6.3 Online teaching readiness across gender, years of teaching experience, and faculty affiliation?

This study revealed that female faculty members are readier than male faculty members when it comes to course design and attitudes towards online learning, a finding that has been corroborated in other studies such as Briggs [45] and Martin et al. [15] where women demonstrated higher self-efficacy in designing and implemented online courses. No significant differences between males and females were identified across other factors. On the other hand, this study showed that more years of teaching experience may result in enhanced pedagogy and course design, a finding that corroborates with Martin et al. [15] and Shea [22].

In terms of faculty affiliation, this study confirmed that online teaching readiness varies across disciplines, a finding that confirms other studies results such as Bolliger et al. [46] and Scherer et al. [47]. Access to Technology was the only factor that was significantly different across faculties especially at the Faculty of Architecture and Fine Arts faculty which offers programs that require art skills such as architecture, visual art, fashion design, and cinema and audio-visual directing. This factor has influenced ALBA faculties' attitudes towards online learning as they scored lower than other faculties. In majors like architecture, research has shown that online studio design should be adopted by schools of architecture as online feedback is a needed skill for architects [64]. Several online technologies for architecture online programs may be adopted: synchronous sessions mostly delivered via Blackboard Collaborate Ultra which allows drawing overlays on top of the students’ work, or asynchronous sessions via ePinup, a private social networking tool that allows students to upload images of their work and receive comments from peers [64]. Such tools are not supported at the institution under study and institutions had to pivot to online teaching without any preparedness. Furthermore, this study shows that faculty lack discipline-specific pedagogical, content, and technological knowledge necessary for a successful online teaching and learning experience [47].

## 7 Limitations

Our study includes several limitations that need to be acknowledged. First, the study was conducted at the breakout of the pandemic and at the beginning of the lockdown which might impact the results had the data been collected pre- or post-pandemic. Second, data was self-reported through an online survey, which might affect the accuracy of the results.

Moreover, this survey was used for data collection at only one academic institution, and thus results may not be generalizable to the entire Lebanese academic population. In addition, the questionnaire used was long and therefore it is preferable to administer it through face-to-face interviews, but in view of the COVID-19 pandemic, the questionnaire was administered online. Furthermore, follow-up interview data is frequently reported together with questionnaire data, which is not the case in our current study.

## 8 Conclusion and recommendations

The aim of this study was to identify factors associated with faculty readiness to teach online in a Lebanese context as well as their overall readiness. The study revealed that faculty have an adequate level of readiness when it comes to online teaching as they possess the minimum requirements in terms of access to hardware (90 %), technology skills (more than 80 %), course design skills (more than 50 %), online course facilitation (70 %), and motivation to advance their technology skills (more than 70 %). Challenges associated with their readiness to teach online included the lack of reliable internet (around 40 %), advanced skills to create interactive online activities (50 % lack this skill), institutional support (30–40 % perceive this as a challenge), The exploratory factor analysis highlighted the existence of five contextual factors informing readiness to teach online, namely technology access and skills, course design, online pedagogy, attitude and institutional support. All five factors were found to reliably describe the components governing faculty's readiness for the transition to online education. The study also revealed that female faculty reported more confidence when it comes to course design. On the other hand, pedagogical, content and technological knowledge seemed to be inadequate in some majors that require art and design skills.

Institutions should move beyond COVID 19 to integrate best practices adopted while teaching online and progress toward a student-centered blended approach of learning. In this respect, the findings of this study imply the need for a more reliable infrastructure for the delivery of online learning in Lebanon and a need for professional development for faculty to create pedagogically and technically enhanced online courses. Furthermore, more emphasis needs to be placed on affective dimensions including sense of belonging in virtual environments [63], anxiety to teach online [61], professional vulnerability [1], and identity disruption [23,25]. It is crucial to expand this research to additional academic institutions and conduct a follow-up study that examines faculty readiness to teach online post-pandemic taking into consideration the experience they have developed and the current economic situation in Lebanon which may be a trigger to disseminate and enhance quality online education.

## Ethics statement

Institutional approval to conduct this study and to disseminate it to the university community was obtained from the office of the Provost. The survey was shared with the faculty through the institution's official email list and via an announcement email sent on behalf of the office of the Provost. By completing the online survey, the faculty had provided their consent to participate in this study.

## Declaration of competing interest

The authors declare that they have no known competing financial interests or personal relationships that could have appeared to influence the work reported in this paper.

## Figures and Tables

**Table:** 

Author(s)	Online Teaching Readiness-Variables measured
Keramati, Afshari-Mofrad, & Kamrani (2011)	Technical, organizational, social
Phan & Dang (2017)	Attitudes, technology competency, pedagogy, training, and time constraint.
Chi (2015)	Teaching and Learning, Social and Student Engagement, Faculty and Technology Support, Course Development and Instructional Design, and Evaluation and Assessment
Gay (2016)	Technical readiness, lifestyle readiness and pedagogical readiness.
Koo (2008)	Time constraint, access to technology, new learning paradigm, collaboration intention, perceived readiness, perceived positive effects of OCL
Soetan & Cocker (2018)	Access of instructor to online technologies: streaming technologies, social media, virtual learning environments (relatively low), Readiness of University instructors in Nigeria to use online technologies in 5 statements (relatively high) a Motivation to use online technology for instructional purposes in 5 statements (relatively high)
Martin, Budhrani & Wang (2019)	Course design, course communication, time management, and technical**,** Attitude and Ability
Pennsylvania State University (2017) https://behrend-elearn.psu.edu/weblearning/FacultySelfAssessment/	Technical competencies, administrative competencies, pedagogical competencies.
University of Toledo (2017). https://www.utdl.edu/lv/assessments/faculty_readiness.html	Basic Technical Skills, Learning Management System (Blackboard) Experience, Course Planning & Time Management, communication, course design
Wright (2014)	Motivation, barriers
Martin, Ritzhaupt, Kumar & Budhrani (2019)	Course design, course communication, time management, and technical competence.
Mercado (2008)	Technology access, technology skills, attitude towards e-learning, institutional readiness
Adhafeeri & Khan (2016)	institutional, ethical, resource support, pedagogical, institutional, management, interface design, technological
Kovanovic et al. (2018)	teaching presence, social presence, cognitive presence 3 factors: course organization and design (sub-component of teaching presence), group affectivity (sub-component of social presence), resolution phase of inquiry learning (sub-component of cognitive presence
Cutri, Mena & Whiting (2020)	Comfort with risk, identity disruption, teaching norms, equity and tenure norms.
Scherer, Howard, Tondeur, & Siddiq (2021)	Technological and Pedagogical Content Knowledge, online teaching presence, Institutional support for online teaching and learning, gender, academic disciplines, previous teaching experiences, culture and innovation.
Cutri & Mena (2020)	Pedagogical, organizational, affective factors

**Table:** 

Faculty readiness competencies	SD	D	N	A	SA
1. I have access to a personal computer or laptop 2. I have access to a printer 3. I have access to a scanner 4. I have access to a webcam and a microphone that I can use for video conferencing 5. I have access to reliable internet connection when needed 6. I can complete basic computer operations 7. I can use basic internet functions 8. I can send and receive emails and open and send email attachments 9. I have the skills to create instructional videos 10. I have the skills to create online tests and assignments 11. I can use web-conferencing tools 12. I can use basic features of Moodle or any other Learning Management System 13. I can use advanced features of Moodle or any other Learning Management System 14. I can provide a comprehensive syllabus that adheres to my institution's policy. 15. I can write measurable learning outcomes and specific course objectives 16. I can design assignments that assess my course learning outcomes. 17. I can revise course content and instructional materials based on student feedback, as needed 18. I can design learning activities that provide students opportunities for interaction in an online environment 19. I am able to develop an online course 20. I lecture to my students for most of the online class session 21. I provide timely and constructive feedback on students' assignments 22. I use non-traditional assessments 23. I send reminders to students about upcoming assignments 24. I can use online discussion as a means of teaching 25. I can use online chat function as a means of teaching. 26. I use a variety of teaching strategies to promote online interaction among my students 27. I can accommodate students that have accessibility issues 28. I can continuously monitor and manage student progress by using course statistics or reports on Moodle 29. I communicate effectively and comfortably in writing using email or text messaging 30. I respond to students' questions promptly 31. I can communicate expectations about my students' online behavior in my course 32. I feel comfortable conveying my personality and/or emotions through speaking 33. I find that online teaching takes more time than face-to-face instruction 34. I am good at organizing teaching materials into modules, weeks, topics, or units 35. I usually schedule time to design the course prior to delivery 36. I have the time to develop an online course 37. I use features in Moodle or another tool in order to manage time 38. I am open to learning more ways in using technology for teaching 39. I believe teaching online offers greater course flexibility for students 40. I believe teaching online diversifies program offerings 41. I believe teaching online offers more opportunities to learn new technologies and improve my teaching 42. I believe online learning is able to promote the students' acquisition of skills 43. I am motivated to teach online 44. I believe my university supports innovative teaching methods 45. I have persons and/or resources at my university who will assist me with any technical problems I might have with my software applications as well as my computer hardware 46. I need more training and guidance to implement online learning in my courses					

**Table:** 

	Variables	Frequency	Percentage (%)
*Age*	21–30	13	6.2
	31–40	84	40
	41–50	53	25.2
	51–60	50	23.8
	>60	10	4.8
*Gender*	Female	115	54.8
	Male	95	45.2
*Faculty*	Fine Arts	15	7.1
	Arts and Sciences	70	33.3
	Business and Management	20	9.5
	Engineering	36	17.1
	Health Sciences	37	17.6
	Medicine and Medical Sciences	2	1.0
	Issam Fares Faculty of Technology	28	13.3
	Saint John of Damascus Institute of Theology	2	1.0
*Rank*	Instructor	72	34.3
	Lecturer	24	11.4
	Senior Lecturer	6	2.9
	Post-Doc	2	1
	Assistant Professor	51	24.3
	Associate Professor	31	14.8
	Full Professor Other	14 10	6.7 4.6
*Academic status*	Part-time	80	38.1
*Teaching experience*	Full time 1–3 years	130 14	61.9 6.7
	4–6 years	34	16.2
	7–10 years	44	21
	Above 10 years	118	56.2
*Online teaching experience*	Less than 6 months	168	80
	6 months-1 year	17	8.1
	More than 1 year	25	11.9

## Appendix A

An Overview of Factors Influencing Online Teaching Readiness Based on the Literature.

## Appendix B

The Survey.

SD: Strongly Disagree D: Disagree N: Neither Agree nor Disagree.

A: Agree SA: Strongly Agree.

## Appendix C

Faculty Demographics.

## References

[1] Cutri R.M., Mena J. (2020). A critical reconceptualization of faculty readiness for online teaching. Dist. Educ..

[2] Quintana C. (March 2020). https://www.usatoday.com/story/news/education/2020/.

[3] Cutri R.M., Mena J., Whiting E.F. (2020). Faculty readiness for online crisis teaching: transitioning to online teaching during the COVID-19 pandemic. Eur. J. Teach. Educ..

[4] Lohr A., Stadler M., Schultz-Pernice F., Chernikova O., Sailer M., Fischer F., Sailer M. (2021). On powerpointers, clickerers, and digital pros: investigating the initiation of digital learning activities by teachers in higher education. Comput. Hum. Behav..

[5] Rajab M.H., Gazal A.M., Alkattan K. (2020). Challenges to online medical education during the COVID-19 pandemic. Cureus.

[6] Easton S.S. (2003). Clarifying the instructor's role in online distance learning. Commun. Educ..

[7] Ko S., Rossen S. (2017).

[8] Khaddage F., Fayad R., Moussallem I. (2020). Online learning and the role of technologies during covid19 pandemic “higher education Lebanon case. Proc. EdMedia + Innov. Learn..

[9] El-Ghali H.A., Ghosn E. (2019). https://www.aub.edu.lb/ifi/Documents/publications/research_reports/2018-2019/20190221_towards_connected_learning_in_lebanon.pdf.

[10] Zgheib G., Al Daia R., Serhan M., Melki A. (2020). Factors influencing students' online learning readiness in a middle eastern higher education institution: implications for online course design. Int. J. e Learn..

[11] Tarhini A., Teo T., Tarhini T. (2016). A cross-cultural validity of the E-learning Acceptance Measure (ElAM) in Lebanon and England: a confirmatory factor analysis. Educ. Inf. Technol..

[12] El Turk S., Cherney I.D. (2016). Perceived online education barriers of administrators and faculty at a US university in Lebanon. Creighton J. Interdisciplinary Leader..

[13] Melki A., Nicolas M., Khairallah M., Adra O. (2017). Information and communications technology use as a catalyst for the professional development: perceptions of tertiary level faculty. Int. J. Educ. Dev. using Inf. Commun. Technol. (IJEDICT).

[14] Cranfield D.J., Tick A., Venter I.M., Blignaut R.J., Renaud K. (2021). Higher education students' perceptions of online learning during COVID-19—a comparative study. Educ. Sci..

[15] Martin F., Budhrani K., Wang C. (2019). Examining faculty perception of their readiness to teach online. Online Learn..

[16] Badiozaman I.F.A. (2021). Exploring online readiness in the context of the COVID 19 pandemic. Teach. High. Educ..

[17] Phan T.T.N., Dang L.T.T. (2016). Teacher readiness for online teaching: a critical Review. Int. J. Open and Distance E-Learn..

[18] Zhen Y., Garthwait A., Pratt P. (2008). Factors affecting faculty members' decision to teach or not to teach online in higher education. Online J. Dist. Learn. Adm..

[19] Wright J.M. (2014). Planning to meet the expanding volume of online learners: an examination of faculty motivation to teach online. Educ. Plann..

[20] Tabata L.N., Johnsrud L.K. (2008). The impact of faculty attitudes toward technology, distance education, and innovation. Res. High. Educ..

[21] Mitchell B., Geva-May I. (2009). Attitudes affecting online learning implementation in higher education institutions. J. Distance Educ..

[22] Shea P., Pickett A., Li C.S. (2005). Increasing access to higher education: a study of the diffusion of online teaching among 913 college faculty. Int. Rev. Res. Open Dist. Learn..

[23] Golden J.E. (2016). http://ezsecureaccess.balamand.edu.lb/login?url=.

[24] Johnson H., Ehrlich S., Watts-Taffe S., Williams C. (2014). Who am I here? Disrupted identities and gentle shifts when teaching in cyberspace. J. Instruct. Res..

[25] Tagg J. (2012). Why does the faculty resist change?. Change.

[26] Kelchtermans G. (1996). Teacher vulnerability: understanding its moral and political roots. Camb. J. Educ..

[27] Bolliger D.U., Halupa C. (2022). An investigation of instructors' online teaching readiness. TechTrends.

[28] Mercado C.A. (December 2008). Fifth International Conference on eLearning for Knowledge-Based Society.

[29] El Feghaly Y., Bou Nader R., Hariri N., Jallouli R., Bach Tobji M.A., Mcheick H., Piho G. (2021). Digital Economy, Emerging Technologies and Business Innovation. ICDEc 2021.

[30] Keramati A., Afshari-Mofrad M., Kamrani A. (2011). The role of readiness factors in E-learning outcomes: an empirical study. Comput. Educ..

[31] Martin F., Ritzhaupt A., Kumar S., Budhrani K. (2019). Award-winning faculty online teaching practices: course design, assessment and evaluation, and facilitation. Internet High Educ..

[32] Paliwal M., Singh A. (2021). Teacher readiness for online teaching-learning during COVID – 19 outbreak: a study of Indian institutions of higher education. Interact. Technol. Smart Educ..

[33] Naylor D., Nyanjom J. (2021). Educators' emotions involved in the transition to online teaching in higher education. High Educ. Res. Dev..

[34] Tondeur J., Scherer R., Baran E., Siddiq F., Valtonen T., Sointu E. (2019). Teacher educators as gatekeepers: preparing the next generation of teachers for technology integration in education. Br. J. Educ. Technol..

[35] Bao W. (2020). COVID-19 and online teaching in higher education: a case study of Peking University. Hum. Behav. Emerging Technol..

[36] Gay G.H.E. (2016). An assessment of online instructor e-learning readiness before, during, and after course delivery. J. Comput. High Educ..

[37] Fayer L. (2014). A multi-case study of student perceptions of online course design elements and success. Int. J. Scholarsh. Teach. Learn..

[38] Tubbs S., Moss S. (2006).

[39] Moore M.G., Keegan D. (1993). Theoretical Principles of Distance Education.

[40] Varvel V.E. (2007). Master online teacher competencies. Online J. Dist. Learn. Adm..

[41] Cross T., Polk L. (2018). Burn bright, not out: tips for managing online teaching. Journal of Educators Online.

[42] Aydin C.H. (2005). Turkish mentors' perception of roles, competencies and resources for online teaching. Turk. Online J. Dist. Educ..

[43] Visser J.A. (2000). Faculty work in developing and teaching web-based distance courses: a case study of time and effort. Am. J. Dist. Educ..

[44] Scherer R., Howard S.K., Tondeur J., Siddiq F. (2021). Profiling teachers' readiness for online teaching and learning in higher education: who's ready?. Comput. Hum. Behav..

[45] Briggs S. (2005). Changing roles and competencies of academics. Act. Learn. High. Educ..

[46] Bolliger D.U., Shepherd C.E., Bryant H.V. (2019). Faculty members' perceptions of online program community and their efforts to sustain it. Br. J. Educ. Technol..

[47] Scherer R., Howard S.K., Tondeur J., Siddiq F. (2021). Profiling teachers' readiness for online teaching and learning in higher education: who's ready?. Comput. Hum. Behav..

[48] Scherer R., Siddiq F., Howard S.K., Tondeur J. (2023). Gender divides in teachers' readiness for online teaching and learning in higher education: do women and men consider themselves equally prepared?. Comput. Educ..

[49] Ball S.J., Lacey C., Woods P. (2012). Teacher Strategies: Explorations in the Sociology of the School.

[50] Scherer R., Siddiq F., Howard S.K., Tondeur J. (2023). The more experienced, the better prepared? New evidence on the relation between teachers' experience and their readiness for online teaching and learning. Comput. Hum. Behav..

[51] Koo A. (2008). Factors affecting teachers' perceived readiness for online collaborative learning: a case study in Malaysia. Educ. Technol. Soc..

[52] Chi A. (2015). http://ezsecureaccess.balamand.edu.lb/login?url.

[53] Gay G.H.E. (2016). An assessment of online instructor e-learning readiness before, during, and after course delivery. J. Comput. High Educ..

[54] Kovanović V., Joksimović S., Poquet O., Hennis T., Čukić I., de Vries P., Hatala M., Dawson S., Siemens G., Gašević D. (2018). Exploring communities of inquiry in massive open online courses. Comput. Educ..

[55] (2023). Faculty Online Teaching Readiness Survey.

[56] (2023). How Ready Are You to Teach Online?.

[57] (2023). Faculty self-assessment: preparing for online teaching, University of Pennsylvania. https://behrend-elearn.psu.edu/weblearning/FacultySelfAssessment/.

[58] Ncube S., Dube L., Ngulube P. (2014). E-learning readiness among academic staff in the Department of Information Science at the University of South Africa. Mediterr. J. Soc. Sci..

[59] Liu L., Jones P.E., Sadera W.A. (2010). An investigation on experienced teachers' knowledge and perceptions of instructional theories and practices. Comput. Sch..

[60] Brennan J., Broek S., Durazzi N., Kamphuis B., Ranga M., Ryan S. (2014).

[61] Bruggeman B., Tondeur J., Struyven K., Pynoo B., Garone A., Vanslambrouck S. (2021). Experts speaking: crucial teacher attributes for implementing blended learning in higher education. Internet High Educ..

[62] Hung M.L. (2016). Teacher readiness for online learning: scale development and teacher Perceptions. Comput. Educ..

[63] Adnan M. (2018). Professional development in the transition to online teaching: the voice of entrant online instructors. ReCALL.

[64] Yu R., Ostwald M.J., Gu N., Skates H., Feast S. (2022). Evaluating the effectiveness of online teaching in architecture courses. Architect. Sci. Rev..

